# Landscape of pediatric cancer treatment refusal and abandonment in the US: A qualitative study

**DOI:** 10.3389/fped.2022.1049661

**Published:** 2023-01-09

**Authors:** Daniel J. Benedetti, Catherine M. Hammack-Aviran, Carolyn Diehl, Laura M. Beskow

**Affiliations:** ^1^ Division of Hematology and Oncology, Department of Pediatrics, Vanderbilt University Medical Center, Nashville, TN, United States; ^2^ Center for Biomedical Ethics and Society, Vanderbilt University Medical Center, Nashville, TN, United States

**Keywords:** bioethics, childhood cancer, abandonment, non-adherence, pediatric oncology, ethics

## Abstract

**Objective:**

To describe United States (US) pediatric oncologists’ experiences with treatment refusal or abandonment, exploring types and frequency of decision-making conflicts, and their impact.

**Study design:**

We conducted exploratory qualitative interviews of pediatric oncologists (*n* = 30) with experience caring for a pediatric patient who refused or abandoned curative treatment. Interviewees were recruited using convenience and nominated expert sampling, soliciting experiences from diverse geographic locations and institution sizes across the US. We analyzed transcripts using applied thematic analysis to identify and refine meaningful domains.

**Results:**

Many oncologists reported multiple experiences with refusal and abandonment. Most anticipated case frequency would increase due to misinformation, particularly on the internet. Interviewees described cases of treatment refusal and abandonment, but also a wider variety of cases than previously described in existing publications, including cases involving: non-adherence; negotiations for different treatments; negotiations for complementary and alternative medicine; delayed treatment initiation; and refusal of a component of recommended therapy. Cases often involved multiple stages or types of conflicts. Recurring patient/family behaviors emerged: clear opposition to treatment from the outset; hesitancy about treatment despite initiating therapy; and psychosocial circumstances becoming an obstacle to treatment completion. Oncologists revealed substantial professional and personal repercussions of these cases.

**Conclusion:**

Oncologist interviews highlight a broad range of conflicts, yielding a taxonomy of treatment refusal, non-adherence and abandonment (TRNA) that accounts for the heterogeneity of situations described. Cases’ complexity and interrelatedness points to a functional model of TRNA that includes families’ behaviors. This preliminary taxonomy and model warrant further research and examination to refine the model and generate strategies to prevent and mitigate TRNA.

## 1. Introduction

In high-income countries like the United States (US), cases of children whose families refuse treatment for favorable-prognosis cancers often garner significant media attention (1–8). For example, a highly publicized 2015 case involved a 17-year-old who was involuntarily hospitalized for longer than 4 months to complete treatment because she was regarded as a significant flight risk, and her prognosis deemed too good to forgo treatment (7). The 2006 case of a 15-year-old whose parents were charged with medical neglect for refusing treatment of a highly curable cancer (9, 10), and the attention it attracted, prompted Virginia to pass “Abraham’s Law” (11), granting children at least 14 years old the right to refuse life-saving treatment under certain conditions.

To date, scholarly publications about such cases predominantly consist of single case descriptions and ethical commentaries (10, 12–17), as well as a systematic review (18) that described patterns in published cases and highlighted important gaps in the literature. The only empirical publications are those focusing on pediatric cancer treatment abandonment in low- and middle-income countries (8, 19–25); a survey of German pediatric oncology programs soliciting the number and details of refusal cases (26); and two quantitative surveys of US pediatricians and pediatric oncologists using hypothetical cases, which asked whether they would pursue court-ordered treatment (27) or support the patient’s refusal (28).

Given significant gaps in the literature, a broader conceptual framework [such as has been developed in other areas of medicine (29)] is needed to help: (1) Define and organize types of conflicts between pediatric oncologists and patient/families; (2) Identify factors that mediate and moderate treatment refusal and abandonment; and (3) Examine interventions to address treatment refusal and abandonment (30). To inform such a framework, we conducted exploratory qualitative interviews with pediatric oncologists who reported personal experiences with such cases. In this analysis, we report their experiences with an emphasis on the first two goals; subsequent analyses will describe management strategies and outcomes of reported cases.

## 2. Materials and methods

### 2.1. Participants

We conducted in-depth interviews with pediatric oncologists in the US. Prospective interviewees were identified through the first author’s professional networks and relationships, as well as nominated expert sampling (31). We endeavored to maximize diversity with respect to gender, race/ethnicity, years of practice, and geographic location. To confirm eligibility, we developed a brief survey eliciting experience with treatment refusal (Table 1).

**Table 1:** Interviewee experiences with case types.

In the past 5 years, have you had first-hand experience with a pediatric oncology case involving any of the following? Check all that apply		
	#	%
Up-front refusal of all recommended cancer-directed therapy	16	53
Up-front refusal of a component of recommended cancer-directed therapy (chemo or surgery or radiation)	17	57
Abandonment of cancer-directed therapy after initial acceptance and receipt of some therapy	18	60
Negotiation of an alternate treatment plan that substantially decreases the likelihood of cure, as defined by the treating physician	10	33
Negotiation for the inclusion of Complementary or Alternative Medicine (CAM) as a patient/parent requirement for acceptance of the recommended therapy	12	40
Delay in the initiation of therapy caused by parental refusal or indecision that causes harm to the child, compromises the likelihood of cure or requires intensification of therapy, as defined by the treating physician	13	43

### 2.2. Instrument development

We developed a semi-structured interview guide focusing on types of conflicts encountered; factors and strategies considered in response; effects of treatment refusal cases, personally and professionally; the role of ethical frameworks and legal requirements; and the resources needed to manage treatment refusal and abandonment cases. After refinements based on pilot testing, the final instrument (available upon request) comprised 14 questions.

The Vanderbilt University Institutional Review Board deemed this research exempt under 45 C.F.R. §§46.104(d)(2)(ii).

### 2.3. Procedures

Interviews were conducted by telephone between May and September 2019 by two experienced team members. At the beginning of each interview, we reviewed a study information sheet and obtained the participant’s verbal agreement to participate and permission for audio recording. Interviews averaged approximately 45 min in length. Participants were offered $100 compensation for their time.

### 2.4. Data analysis

We uploaded professionally transcribed interviews into qualitative research software NVivo 12 for coding and analysis using standard iterative processes (32, 33). A subset of the data are presented here, with further analyses planned for future manuscripts.

See Appendix 1 for further methodologic details (34).

## 3. Results

### 3.1. Participant characteristics

We interviewed 30 pediatric oncologists representing a range of perspectives based on institutional volume, practice location, and time in practice (Table 2). Further, in the pre-interview survey, participants reported a range of experiences with different types of cases (Table 1).

**Table 2:** Participant characteristics^a^ (*n* = 30).

	*n*	(%)
**Years in practice post-fellowship**		
0–5 years	9	30
6–10 years	7	23
11–15 years	5	17
16–20 years	3	10
21–25 years	1	3
>26	3	10
Unknown	2	7
**Gender**		
Female	16	53
Male	14	47
**Race^b^**		
White	23	77
African American	0	0
Asian	5	17
Native Hawaiian or Pacific Islander	0	0
American Indian or Alaska Native	0	0
Prefer not to answer	0	0
Unknown	2	7
**Ethnicity**		
Hispanic/Latino	0	0
Not Hispanic/Latino	28	93
Unknown	2	7
**Regional distribution**		
Northeast, New England	4	13
Northeast, Middle Atlantic	3	10
South, South Atlantic	5	17
South, East South Central	3	10
South, West South Central	0	0
Midwest, East North Central	6	20
Midwest, West North Central	3	10
West, Pacific	3	10
West, Mountain	3	10
**Institutional volume (new cancer diagnoses per year)**		
<100	10	33
100–200	9	30
>200	9	30
Unknown	2	7

^a^ All responses were self-reported by participants on the pre-interview survey.

^b^ For “Race” participants were invited to check all that apply.

Narrative segments presented here (with participant IDs) are exemplary of frequently mentioned ideas, with additional illustrative quotations in Table 3.

**Table 3:** Themes with illustrative quotes.

Theme	Subtheme	Representative quote
Frequency and Incidence of Treatment Refusal	Multiple experiences with Treatment Refusal	In 17 years, it’s happened maybe a half dozen times, I would estimate. (ID_16) I can actually think of two, three, four, five, six cases where I’ve been involved as the attending for patients who’ve refused care. (ID_18)
	Infrequent, yet universal experience	I think they’re rare, but everybody has a story about either noncompliance, or refusal. Every provider has had that happen some time. (ID_15)
	Frequency estimates	We probably have one every one or two years, so not often. (ID_14) I don’t know, a couple times a year. I personally am having these discussions two or three times a year. (ID_19) It’s at least maybe between 3% and 5% of the cases we have. At least we see families thinking about alternatives and [start] to get the sense that they are thinking about refusing. (ID_22)
	Expected increase in frequency	I would say the problem is getting worse… (ID_21) I think it will definitely increase. (ID_26)
Reasons for Increased Treatment Refusal	Internet & misinformation	You’d have to wonder if it is more common because of the internet and people’s easy access to alternative information, some of which is good but a lot of which is not. (ID_06) There are a lot of voices on the internet who champion some fringe or less well studied treatments, and parents who are in very emotional, very stressful situations have an easier time finding somebody who has more outside the box ideas shall we say. (ID_21) The internet age and the proliferation of knowledge on the internet, the propagation of the whole patient empowerment movement, Facebook blogs, online forums, these drive a lot of this and… these parents think they know better than the doctors because they have read on the internet and they’ve spoken to other parents. (ID_26)
	Diminishing trust in medicine	In the age of the internet, and Facebook, and mom groups, and parent groups, there’s a greater dissemination of false information, which leads to, I think, greater questioning of the medical establishment overall. (ID_08) In general the whole unwavering trust in the medical field and physicians is decreased compared to 20 years ago. The physician was the epitome of … the honorable person in a community. I think that’s less and less how people view things. (ID_20)
	Knowledge as asset and risk factor	It’s a good thing for families to know and understand that there’s different opinions, and they should feel empowered to bring those things up and ask those questions… I’ve seen this more and more over the last couple of years, in part because families are so educated, and have access to so much information. I think that’s really beneficial to our patients, because families have access to understand what’s happening with their child… But I do think there’s a lot of misinformation available online. So I think cases have increased… I think that’s going to continue and potentially at higher rates. As oncologists we are going to have to learn how to navigate that. (ID_28)
Types of Refusal Cases	Distinction between refusal/abandonment, and non-adherence	We see non-adherence fairly often. We don’t see abandonment very often, and we don’t see flat-out refusal very often. People who are not compliant with their appointments or their medications… is not uncommon. (ID_25) I was viewing this more as outright refusal of upfront therapy or abandonment of therapy, but if I expand that to noncompliance with recommended therapy, which is a little bit different… (ID_26)
Patient/Family Behaviors	Opposition	There was the upfront, “We don’t think we want to treat…” (ID_09) In that initial conversation I could tell that something was just not connecting. There was a lot of skepticism from the family that the child actually had leukemia. They thought that we were lying to them and that we were trying to do terrible things with their child to experiment or something. (ID_29)
	Hesitancy	His treatment was going to require chemotherapy and surgery and radiation. The family didn’t want the radiation… At the very beginning when we were talking about the overall treatment course, they said “that’s not something we’re going to want.” (ID_07) The father of a patient with leukemia, did not want to consent for therapy… [He wanted to do] less therapy, and for not as long. (ID_15)
	Psychosocial circumstances	It turned out that he was pretty deeply involved with several friends that were having very difficult situations where he felt like he needed to be present for them, and getting treated for cancer took him away from where he felt he was needed… I think in his heart he knew that this was really best for him, to get his treatment, but I just think there were so many barriers. (ID_11) I think, this patient had lots of things stacked against him in terms of not having much family support. They were very low income, poor transportation. It was kind of like, right now there about to be evicted from their apartment. They were kind of living on the edge to begin with, and this was just kind of the straw that broke the camel’s back. (ID_13)
Patterns	Alternative medicine	They have surrounded themselves with people that have told them to be wary and not to believe in standard medicine, that believe that natural treatments rule the day over what evidence-based treatments were. (ID_01) We don’t trust your medicine… We want to put our faith in prayer and herbal [treatments]. (ID_09)
	Socioeconomic obstacles	I’ve noticed that it’s families with very limited resources who aren’t very medically informed … it’s often families where the parents are working multiple jobs, who have lots of kids in the home, who have a lot of other priorities and constraints in their life that they can’t pay that degree of close attention to their child with cancer that we would ideally like. (ID_10)
	Distrust/mistrust of US healthcare systems	Due to a long history stuff that people went through and were taken advantage of, there’s that sense of distrust [in] the African American community… I have found that amongst many of our African American patients, that initial, ‘we assume that you are doing the right thing for our child,’ is not always there. (ID_05) This is very anecdotal, but it seems to me that it has happened more often in people of other cultures, and when I say ‘other culture,’ that could be ethnic or another country or even another faith… I think what comes along with this as an inherent distrust of the system. (ID_14)
	Education, low or high	Sometimes it’s people that are very uneducated that are very distrustful of medicine. Probably more commonly, even, it’s [families] that are pretty educated, but just have surrounded themselves with people that have told them to be wary and not to believe in standard medicine. (ID_01)
	Hodgkin Lymphoma	If you Google this topic, the most common disease where this comes up of refusal of treatment is in Hodgkin’s by far. I think it’s because patients don’t have symptoms until they’re dying… (ID_02)
	Amish	It’s probably the uninsured Amish population that we encounter more. And they seem to be very concerned about payment because they don’t accept government insurance. And maybe also culturally have more of a naturopathic history to their elder population. So I think that they’re more inclined to… abandon treatment. (ID_16)
Impact on Pediatric Oncologists	Difficult	I can describe it best just by the emotions I felt. I felt angry. I felt, of course, stressed out. It was incredibly impactful on a personal level and professional level as well because there was just a lot of layers to it. The feelings I had about them refusing therapy, the feelings I had about being fired as their physician, the feelings I had about their disbelief of the medical literature and their belief that these other methods, that I knew were not based in any sort of reality, were going to help their child.… It was overwhelming in a lot of ways. (ID_26) It was tough. It was really, really difficult. I didn’t go down without a fight. I had many conferences, many conversations, you know, joint conferences with the orthopedic surgeon, had our psychologist talk to her, had our palliative care team talk to her, it was difficult for me and I struggled a lot with it. (ID_27)
	Frustration	It was very frustrating to me how educated people could, in my mind, in my view, make such an uneducated ill-informed decision. (ID_04) Frustrated… I think all of us are very frustrated because the child is otherwise completely healthy and normal. Has no other neurologic or otherwise sequelae from this. And because we know what this looks like when it grows… You feel stuck. And kind of helpless in a sense that you see what’s going to happen and you can’t force someone’s hand in this case very easily. (ID_05)
	Time	I had spent hundreds of hours with this family talking things through. These cases are hard in a lot of ways, but one of them is just the sheer amount of hours that you spend talking about it with the family, with other [medical providers], trying to figure it out, wrestling with it, staying up at night, thinking about it. So many hours. (ID_24)
	Feelings of failure	In oncology, we have a lot of difficult conversations with families and building trust, and teaching families about what’s going on is a big part of what we do. So it definitely felt like a failure to not be able to bridge that with them. (ID_30)
	Sadness & Distress	Ugh, it was terribly upsetting… this was many years ago and I remember it vividly and especially, I think the part that upset me the most was that the child was in between us. The child was like, ‘you’re evil. My mom says you’re the devil.’ I want the child to always be with their parents, right? That’s really important. They’re going to have a relationship with me for a year. They’re going to have a relationship with their mother forever. It is really important that they trust their parents and feel their parents are working in their best interest. So, it is never to my advantage to somehow try and interfere with that and say, ‘your mom’s wrong.’ So, that was really hard. (ID_23)
	Fear for safety	People were calling our department at times and making threats. Our security had to notify the local police and they would drive by my house extra. (ID_02)
	Positive impact	I felt like I learned a lot from it. I learned how to kind of…How this whole process works and what was needed in terms of developing kind of as detailed and as compelling an argument as possible in favor of what we were proposing, and when that didn’t work kind of how to come up with a kind of compromise that we still felt gave this patient the best possible survival, while they kind of avoiding something that really would have been traumatic… So ultimately I felt like it was a really educational and it was important to my development as a physician and understanding the processes in place that supports important decisions that we make and that allow us to deal with these kind of challenging situations with families. (ID_22)

### 3.2. Frequency of treatment refusal cases

Although we asked interviewees to describe a single treatment refusal case, many noted they had encountered multiple cases over their careers. Most used general terms to describe the incidence of treatment refusal, but some offered numerical estimates such as: “not often… probably one every one to two years” (ID_14), “two to five times a year” (ID_26), and “between three to five percent of cases” (ID_22). Most interviewees observed that, while these cases are relatively uncommon, they expected most oncologists would encounter them at some point.

When asked whether they anticipated changes in the frequency of treatment refusal cases, most thought it would increase. Many cited the internet and “misinformation available online” (ID_28) as reasons, while others described a societal trend of diminishing trust in medicine and “greater questioning of the medical establishment overall” (ID_08).

### 3.3. Types of treatment refusal cases

When asked to describe a specific case of treatment refusal, interviewees described 37 distinct cases. Their narratives highlighted the complexity of these cases, with each following a unique path from diagnosis to conclusion, and many involving multiple stages of conflict. We grouped the cases into 7 categories (Table 4), including upfront refusal of all recommended therapy, upfront refusal of a component of recommended therapy, non-adherence, abandonment, negotiation of an alternative to recommended therapy, negotiation for inclusion of complementary and alternative medicine along with recommended therapy, and delays in the initiation of treatment. Some interviewees also described patients who completed treatment but missed visits to monitor for recurrence.

**Table 4:** Categories of cases described by pediatric oncologists.

Case type	Defining feature(s)	Example (s)	Number
Treatment Refusal	Non-initiation of treatment Refuses all cancer-directed treatment	Open about refusal Seek ‘natural’ alternatives Denial of cancer diagnosis	18
Partial Refusal	Accepts some cancer treatment Refuses one modality (e.g., surgery, radiation)	Refuses amputation Doesn’t believe radiation is necessary	5
Negotiation	Negotiates alternative treatment plan Negotiates inclusion of complementary or alternative medicine	Wants fewer cycles, or not all of the chemo agents Trial of CBD or Vitamin C therapy before chemo	5
Delay	Initiation of therapy is delayed Comply with workup	Seek multiple opinions Miss or delay staging scans	1
Non-Adherence	Initiates therapy Misses or delays appointments Misses treatments	Poor adherence to oral chemotherapy Attribute to external factors, such as economic obstacles	3
Abandonment	Initiate therapy but discontinue	Blame toxicity Seek “natural” alternatives	17
Lost To Follow-Up	Complete therapy Miss or cancel surveillance visits/scans	Difficult to contact May feel fine Attribute to socioeconomic factors	2

As conveyed by these categories, many interviewees distinguished between cases of non-adherence and treatment refusal or abandonment:

*I haven’t had a lot of patients completely abandon ship, especially once they’ve started, and treatment refusal I think is pretty rare for all of us. We maybe have one or two patients a year who completely refuse chemo… The treatment non-adherence, like not completely taking therapy that’s prescribed, I encounter that every day*. (ID_10)

### 3.4. Recognizing the problem

Interviewees revealed the varied circumstances by which they became aware their patients were unwilling or unable to complete recommended treatment. Some families made their opposition to treatment clear upon receiving the diagnosis and treatment plan. Interviewees described “skepticism from the family that the child actually had [cancer]” (ID_29), families that “really did not trust doctors” (ID_14), and others who “worried about side effects” (ID_09) and preferred to try alternative treatments.

Other interviewees described families who initiated therapy but expressed hesitancy about whether it was necessary, or whether the entirety of the proposed treatment was appropriate. Some opposed one component of multimodal treatment saying it was “not something [they] were going to want” (ID_07), while others negotiated for “less therapy, for not as long” (ID_15). Many families asked about “alternative approaches” (ID_17), and whether alternative medicine “could be used instead of traditional chemotherapy to cure [the cancer]” (ID_08).

*I think they were already a little bit skeptical… a little bit on the fence from the outset about giving him chemotherapy. They very much viewed it as a toxin, and just believed there were natural means that could cure him… They agreed to go forward with the chemo on, I think, a hesitant basis and then once he had this side effect, this toxicity, it pushed it over the edge and I think confirmed their misgivings that they had from the beginning*. (ID_26)

Finally, some families never questioned the necessity or appropriateness of treatment; instead, psychosocial obstacles eventually proved to be insurmountable for the patient to complete intended treatment. This included families who were “living on the edge to begin with, [and cancer] was the straw that broke the camel’s back” (ID_13). Some interviewees highlighted socioeconomic obstacles that impact families’ “compliance and wanting to stop [treatment]” (ID_17). They cited families with “low income”, “poor transportation” (ID_13), “single parents without money who need to work” (ID_11), and “parents [who] are working multiple jobs” (ID_10) as being at risk.

### 3.5. Patterns of patient and family behaviors

When asked to describe patterns or predictors that helped identify those at risk for treatment refusal and abandonment, many interviewees described families “asking to integrate… alternative medicine practices into their oncology care” or even “requesting complementary and alternative approaches instead of standard oncology care” (ID_07). Proposed alternatives included “holistic and naturopathic medicine” (ID_07), ranging from “high-dose Vitamin-C” (ID_29) and “herbal treatments” (ID_09) to “CBD oil or medicinal marijuana” (ID_08). Other common proposals included faith-based healing, reflecting beliefs that “God was going to cure” (ID_23) their child through “prayer” (ID_09) and “holy water” (ID_20).

Many cited distrust/mistrust of US healthcare systems as a common thread. Several described general trends of declining trust in “the medical establishment” (ID_01) and increasing “distrust of authority in our culture” (ID_29). Others referenced “inherent distrust of the [healthcare] system” (ID_14) from members of minority and marginalized populations, meaning the “initial, ‘we assume that you are doing the right thing for our child’, is not always there” (ID_05).

Some interviewees reported opposition from families at the extremes of educational backgrounds, with “people that are very uneducated” (ID_01), as well as “very educated families that do all of the research” (ID_05) frequently pushing back against recommendations. Interviewees commonly described families having more access to medical information (vis-à-vis the internet) as both an asset and a risk factor for treatment refusal.

*It’s a good thing for families to know and understand that there’s different opinions, and they should feel empowered to bring those things up and ask those questions… I’ve seen this more and more, in part because families are so educated, and have access to so much information… That’s really beneficial, because families understand what’s happening with their child… But there’s a lot of misinformation available online. As oncologists we are going to have to learn how to navigate that*. (ID_28)

A few surmised that children with Hodgkin Lymphoma are a higher risk of treatment refusal because most “feel pretty good when they’re diagnosed” (ID_04) and “don’t have symptoms until they’re dying” (ID_02), making it difficult to convince the family that the side effects of chemotherapy are justified.

Finally, multiple interviewees reported that Amish families seemed more likely to abandon treatment, either because of “concerns about payment” or because of cultural interest in “more of a naturopathic” approach (ID_16).

### 3.6. Impact of treatment refusal cases on pediatric oncologists

Many interviewees reported they had “struggled a lot” (ID_27) with these cases that “kept [them] up at night” (ID_03), describing how difficult and “overwhelming” (ID_26) it was at the time.

They frequently described feelings of frustration, including bringing “that frustration home” (ID_10). For some, the frustration was that such “educated people could… make such an uneducated, ill-informed decision (ID_04). Other frustrations were at not being able “to get through [to them] and help” (ID_06).

Many emphasized the “inordinate amount of time” (ID_4) and resources dedicated to these cases. One interviewee spent “hundreds of hours with [the] family talking things through,” in addition to “so many hours” spent “talking with other medical providers… and staying up at night thinking about it” (ID_24).

Some referenced feelings of failure or “self-doubt” (ID_3) at not being able to “build trust” (ID_30) and convince a family to agree to treatment. Because oncologists “have a lot of difficult conversations with families and building trust and teaching families… is a big part of what [oncologists] do” (ID_30), a “broken relationship is a failure… to figure out how to partner with that family” (ID_07).

Some interviewees were “obviously very sad for the high likelihood of losing the patient” (ID_20), and also distressed about the impact on the family unit, such as having parents divided about the child’s treatment, or that the disagreement suggested to the child that their parents might not be “working in their best interest” (ID_23).

In rare cases, interviewees described fear for the safety of the medical team: “People were calling our department at times and making threats. Our security had to notify the local police and they would drive by my house” (ID_02). One recounted a parent encounter involving immediate physical danger:

*He kind of blocked the door, [saying] something along the lines of, ‘How do you sleep at night? How do you look in the mirror? I hope you're proud of yourself’, sort of this, ‘you won and at what cost, you’re a terrible person.’ Then he, kind of blocking the door, stood up and advanced on me. I remember literally thinking only, ‘How do I get out of this room?’ I remember feeling so afraid for my own safety*. (ID_07)

## 4. Discussion

The majority of US children diagnosed with cancer will be cured of their disease (35). Unfortunately, not all patients complete optimal treatment due to treatment refusal or abandonment. Anecdotally, treatment refusal has long been discussed between colleagues, with cases only rarely published in the medical literature. A systematic review emphasized the significant gaps and publication bias, and highlighted the need to report and study effective strategies and solutions to these conflicts (18).

This exploratory study sought to begin addressing this need by eliciting US pediatric oncologists’ experiences managing treatment refusal cases. One major goal was to examine the types of cases experienced, to help generate a taxonomy. Multiple publications emphasize the importance of adopting consistent and precise terminology to identify and document cases of incomplete treatment, ascertain underlying causes, and devise solutions (30, 36, 37). Referencing “semantic chaos”, Weaver et al. propose an algorithm to distinguish categories of treatment incompletion across chronic conditions (including cancer), noting the need for “disease- and context-specific” refinement (30). Most US cases in the literature are either upfront refusal of treatment (*Refusal*) or discontinuation of treatment (*Abandonment*). Some of our interviewees viewed *non-adherence* as a separate, unconnected problem from *refusal and abandonment*. However, the complexity of cases we learned about suggests that while *non-adherence* is a distinct concept [and there are diverse causes or subcategories that warrant different interventions (30)], it is not disconnected from refusal and abandonment. Multiple cases where non-adherence preceded abandonment indicates that they must coexist in a comprehensive functional model, and that a taxonomy based on a false separation between non-adherence and refusal and abandonment would result in an over-simplified model. Our data suggest that these conflicts may be more accurately termed treatment refusal, non-adherence, or abandonment (TRNA).

**Figure 1:**
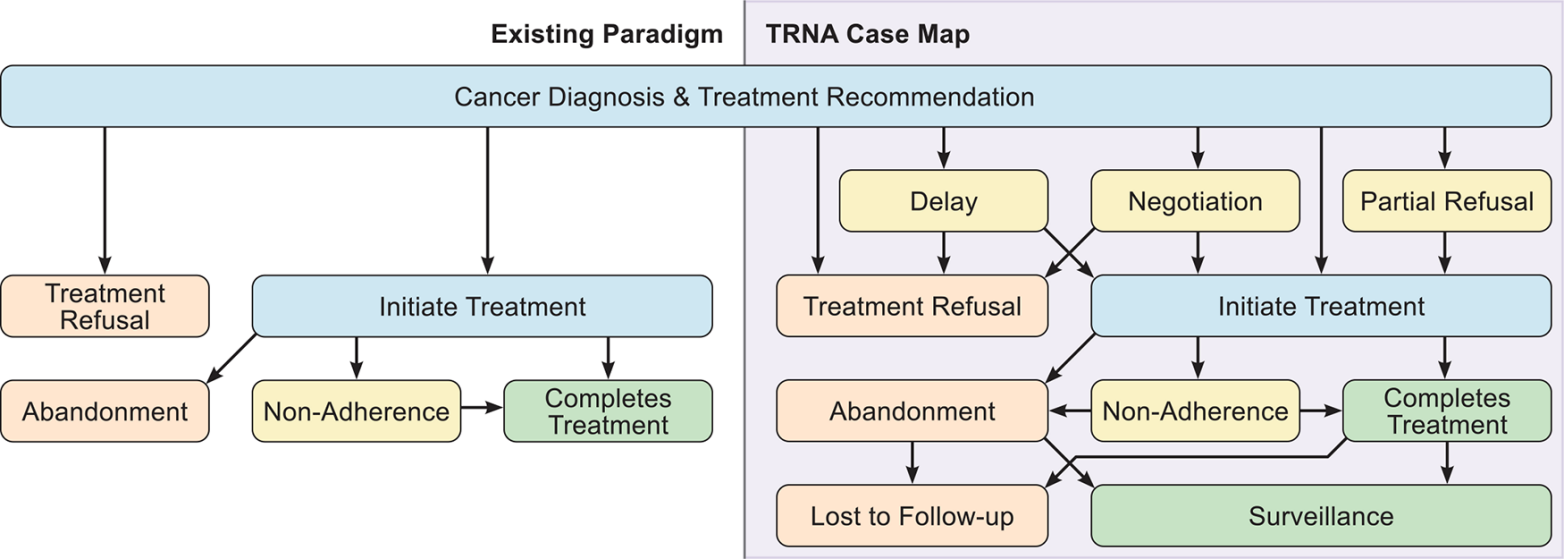
TRNA case map.

Further, our interviewees described complicated, multifaceted experiences that require a nuanced model to account for the full range of conflicts that may preclude receipt of recommended treatment. Many cases included multiple conflicts during the arc of the patient’s cancer diagnosis and treatment. Some patients refused treatment upfront, eventually initiated therapy, only to ultimately abandon treatment. Other patients were non-adherent to treatment, before eventually abandoning therapy altogether. The TRNA case map derived from our interviewees’ cases (Figure 1) depicts this complexity and interconnectedness. This taxonomy should provide for more accurate classification of conflicts, an important precursor to examining interventions that might mitigate or overcome TRNA (30, 36).

**Figure 2:**
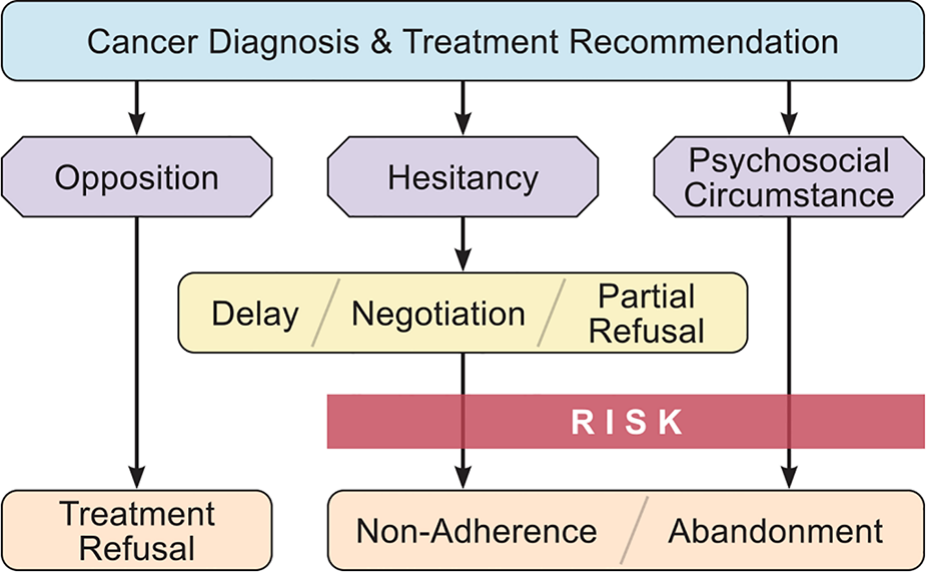
Functional model of TRNA.

A functional model of TRNA would ideally identify patients at risk for non-completion of treatment, and direct oncologists to a set of interventions targeted to their particular circumstance (30). Across our interviewees, three main patterns common to TRNA cases emerged in our analysis. The first involved families with immediate and persistent opposition to cancer treatment. Some denied the child had cancer, while others were opposed to conventional treatments (e.g., radiation, chemotherapy). These cases of *Treatment Opposition* were apparent from the beginning of the relationship with the patient, akin to parents with firm opposition to childhood vaccination (38). A second pattern involved families hesitant about the recommended treatment. Some initially opposed it or questioned the length or necessity for all components, yet ultimately initiated (if hesitancy was apparent upfront) or continued treatment (if hesitancy was expressed mid-treatment). These *Treatment Hesitant* parents may be similar to vaccine hesitant parents who require effective communication strategies to agree to childhood immunizations (38, 39). The third pattern involved families who had *Psychosocial Circumstances* that became significant impediments to completing treatment, resulting in either non-adherence or abandonment. Naturally these circumstances may co-exist within a case where a family opposes or hesitates about treatment, however in some cases they appeared to be the sole factor in treatment non-adherence or abandonment. These three patterns overlap significantly with core issues that have been described as occurring in difficult relationships between pediatric oncologists and parents of children with cancer: problems of connection and understanding, confrontational parental advocacy, mental health issues, and structural challenges to care (40). We combined these patterns with the taxonomy of conflicts to create a functional model for future study and guidance for pediatric oncologists (Figure 2).

A second major goal of our study was to describe the frequency and impact of these cases. While treatment refusal is generally considered to be rare, our interviewees commonly reported multiple experiences with TRNA and the considerable impact it had on them. Some cases demanded substantial professional resources and time, and many burdened oncologists with feelings of distress, failure, and even fear of physical safety.

Our study was exploratory and descriptive in nature, and utilized a purposive recruitment strategy intended to gather a range of experiences reflecting the diversity of pediatric oncology practices in the US. Our findings may be specific to the US context (and possibly other high-income countries) and not fully applicable to pediatric oncology care and treatment incompletion in other settings. Further, given time and resource constraints, our interviews with thirty pediatric oncologists focused in depth on one case they had experienced; our results do not encompass every TRNA case they had encountered or that other oncologists may encounter. While participants were diverse with respect to geography, gender, and practice experience, most self-reported their race/ethnicity as non-Hispanic white or Asian. It is possible that pediatric oncologists from historically marginalized communities might have different experiences and viewpoints on some cases of TRNA, and exploring their perspectives is an important area for future study. Even so, because this study constitutes the only systematic examination of unpublished cases, our taxonomy and functional model provide an important step toward better understanding TRNA.

We carried out these interviews in 2019, prior to the COVID-19 pandemic. Despite changes in the socio-political environment during these years, we believe our results reflect a taxonomy of cases that endures across time. While the pandemic has led to delays in pediatric cancer diagnoses (41–44), to our knowledge no studies describe changes in attitudes toward pediatric cancer treatment. If anything, the seemingly widespread embrace of unproven, unscientific COVID-19 treatments likely reflects increasing skepticism of science, which we anticipate could result in more frequent TRNA cases. Furthermore, the socioeconomic stressors of the pandemic, such as virtual schooling and lost jobs, may exacerbate the psychosocial conditions that increase the risk of non-adherence and abandonment. Further research will be needed to explore any shifts in trends over time.

## 5. Conclusion

In the analysis presented here, we characterized the landscape of TRNA cases experienced by our interviewees. Our findings suggest that pediatric oncologists will need to navigate TRNA multiple times during their professional careers, and to cope with potentially profound personal and professional impact. We hope the proposed taxonomy and functional model serves as a foundation for ongoing conceptual and empirical work to understand and address TRNA. Future examination and analyses of our data are expected to shed light on some of the compromises, solutions, and strategies interviewees employed to overcome hesitancy, and the factors that went into their decisions about whether and when to involve the judicial system.

## Data Availability

The datasets generated and analyzed in this study are not publicly available due to privacy and confidentiality considerations, but are available upon reasonable request from qualified researchers conducting IRB approved studies that fall within the scope of the study purpose and data use described to interviewees at the time of participation. Requests to access the datasets should be directed to Daniel Benedetti, daniel.benedetti@vumc.org.
