# Corrigendum: Landscape of pediatric cancer treatment refusal and abandonment in the US: a qualitative study

**DOI:** 10.3389/fped.2024.1386784

**Published:** 2024-02-26

**Authors:** Daniel J. Benedetti, Catherine M. Hammack-Aviran, Carolyn Diehl, Laura M. Beskow

**Affiliations:** ^1^ Division of Hematology and Oncology, Department of Pediatrics, Vanderbilt University Medical Center, Nashville, TN, United States; ^2^ Center for Biomedical Ethics and Society, Vanderbilt University Medical Center, Nashville, TN, United States

**Keywords:** bioethics, childhood cancer, abandonment, non-adherence, pediatric oncology, ethics

A Corrigendum on Landscape of pediatric cancer treatment refusal and abandonment in the US: a qualitative study By Benedetti DJ, Hammack-Aviran CM, Diehl C, Beskow LM. (2023) Front. Pediatr. 10:1049661. doi: 10.3389/fped.2022.1049661

In the published article, there was an error in including the **Acknowledgements** statement. The statement “We thank Kate Brelsford for expertise and assistance in developing the interview guide and conducting interviews.” should not be included.

The authors apologize for this error and state that this does not change the scientific conclusions of the article in any way. The original article has been updated.

