# Supplementary material for: Landscape of pediatric cancer treatment refusal and abandonment in the US: A qualitative study
Source: Front Pediatr. 2023 Jan 9;10:1049661. doi: 10.3389/fped.2022.1049661 (PMC9869139; doi:10.3389/fped.2022.1049661)
Supplement: Supplementary file 1 [file Table1.docx]

**Appendix 1. Consolidated Criteria for Reporting Qualitative Studies (COREQ)**

| **RESEARCH TEAM AND REFLEXIVITY** | |
| --- | --- |
| **PERSONAL CHARACTERISTICS** | |
| 1. interviewer: Which authors conducted the interviews? | The interviews were conducted by Catherine Hammack-Aviran (author) and Kate Brelsford (acknowledged) under the leadership of the Principal Investigator Daniel Benedetti (author) and Senior Investigator Laura Beskow (author). |
| 2. credentials: What were the researcher’s credentials? | Daniel Benedetti, MD, MA [DB] (author); *Principal Investigator*; Assistant Professor of Pediatrics, Pediatric Hematology/Oncology, Core Faculty, *Center for Biomedical Ethics & Society*; man; pediatric oncology, bioethics.  Laura Beskow, MPH, PhD [LB] (author): *Senior Investigator;* Professor of Health Policy; Ann Geddes Stahlman Chair in Ethics and Society, *Center for Biomedical Ethics & Society;* woman; empirical bioethics  Kate Brelsford, MPH, PhD [KB] (acknowledged); Core Faculty, *Center for Biomedical Ethics & Society*, Research Assistant Professor of Health Policy; woman; qualitative research, empirical methodology, medical anthropology  Catherine Hammack-Aviran, MA, JD [CHA] (author); Core Faculty, *Center for Biomedical Ethics & Society*, Associate in Health Policy; woman; medical law, bioethics, qualitative research  Carolyn Diehl, MS [CD] (author); Program Manager, *Center for Biomedical Ethics & Society,* woman; qualitative research |
| 3. occupation: What was their occupation at the time of the study? |  |
| 4. gender: [What are the researchers’ genders?] |  |
| 5. experience and training: What experience or training did the researcher have? | DB has over five years as a practicing pediatric oncologist and clinical bioethicist. He has formal education and training in bioethics, including qualitative research methods.  LB has over twenty years of experience conducting qualitative and quantitative empirical research on ethical issues in biomedical research and the practice of medicine and public health.  KB has over fifteen years of experience in qualitative research, including the conduct of semi-structured interviews and qualitative coding and analysis.  CHA has over ten years of experience in social science research, including the conduct of semi-structured interviews and qualitative coding and analysis.  CD has over 5 years of qualitative human subject research experience including conducting interviews, coding, and analysis |
| **RELATIONSHIP WITH PARTICIPANTS** | |
| 6. relationship established: Was a relationship established prior to study commencement? | No relationship was established between an interviewee and interviewer prior to study commencement. |
| 7. participant knowledge of the interviewer: What did the participants know about the researcher? *(e.g., personal goals, reasons for doing the research)* | Prospective participants were provided with written information about the funding source, the overall purpose of the study, and the specific goals of the interview. |
| 8. interviewer characteristics: What characteristics were reported about the interviewer/facilitator? *(e.g., bias, assumptions, reasons and interests in the research topic)* | At the start of each interview, all participants were informed that their interviewer was not medically trained, in the context of requesting lay explanations of any complex medical information. |
| **STUDY DESIGN** | |
| **THEORETICAL FRAMEWORK** | |
| 9. methodological orientation and theory: What methodological orientation was stated to underpin the study? *(e.g., grounded theory, discourse analysis, ethnography, phenomenology, content analysis)* | We used an over-arching grounded theory research methodology. Within the overall framework, we employed an applied thematic analysis (including constant comparative analysis) to identify and refine meaningful domains. |
| **PARTICIPANT SELECTION** | |
| 10. sampling: How were participants selected? *(e.g., purposive, convenience, consecutive, snowball)* | Purposive and referral sampling, as described under *Methods: Participants.* |
| 11. method of approach: How were participants approached? *(e.g. face-to-face, telephone, mail, email)* | Prospective participants were informed of the opportunity to participate in this study via email initially distributed by DB to one or two individuals within pediatric hematology/oncology programs around the United States. DB encouraged recipients to share study information with colleagues at their institution, who could complete a REDCap survey to express interest and determine eligibility. |
| 12. sample size: How many participants were in the study? | n = 30   - 18 participants from nominated expert sampling - 12 participants from professional networks |
| 13. non-participation: How many people refused to participate or dropped out? Reasons? | Among the 42 individuals who contacted us (via completion of REDCap survey) to express interest in participating:   - 4 were not evaluable due to incomplete survey responses - 0 were eligible but declined to participate - 6 were invited to schedule an interview but did not respond to the invitation - 2 agreed but failed to respond to our attempts to schedule an interview - 0 scheduled an interview but did not participate (*i.e.*, “no-shows”)   No individual failed to complete an interview in progress (*i.e.*, no one dropped out), and no completed interviews were omitted from the dataset. |
| **SETTING** | |
| 14. setting of data collection: Where was the data collected? *(*e*.g., home, clinic, workplace)* | Interviews were conducted by telephone. |
| 15. presence of non-participants: Was anyone else present besides the participants and researchers? | No |
| 16. description of sample: What are the important characteristics of the sample? *(e.g., demographic data, date)* | The sample is described in detail under *Methods: Participants* and under *Results: Participant Characteristics* (Table 1). |
| **DATA COLLECTION** | |
| 17. interview guide: Were questions, prompts, guides provided by the authors? Was it pilot tested? | The interview guide (available upon request) was developed by the authors under the direction of LB. It was pilot tested with 3 individuals. |
| 18. repeat interviews: Were repeat interviews carried out? If yes, how many? | No interviews were repeated. |
| 19. audio/visual recording: Did the research use audio or visual recording to collect the data? | With each participant’s permission, all interviews were digitally audio-recorded. |
| 20. field notes: Were field notes made during and/or after the interview or focus group? | Yes; interviewers took handwritten notes directly onto interview materials designated for each participant throughout the interview, as well as additional post-interview contextual notes when relevant. |
| 21. duration: What was the duration of the interviews? | Interviews ranged from 21 – 71 minutes in length. On average, each interview lasted approximately 45 minutes. |
| 22. data saturation: Was data saturation discussed? | This was an exploratory study not designed to achieve thematic saturation per se, but rather to begin elucidating the types of TxA cases that pediatric oncologists encounter. In discussing these cases, interviewees described a range of events, strategies, and reactions that we captured in multi-level coding. No new themes emerged after the 25th interview. |
| 23. transcripts returned: Were transcripts returned to participants for comment and/or correction? | No |
| **ANALYSIS AND FINDINGS** | |
| **DATA ANALYSIS** | |
| 24. number of data coders: How many data coders coded the data? | Three (3) [DB, CHA, CD] |
| 25. description of the coding tree: Did authors provide a description of the coding tree? | Major and minor themes are identified within headings and subheadings. |
| 26. derivation of themes: Were themes identified in advance or derived from the data? | Themes were derived from the data.  All members of our interdisciplinary team—with expertise in law, qualitative research, theoretical bioethics, applied bioethics, empirical bioethics, and pediatric oncology—reviewed all transcripts (n=30). Each team member independently reviewed transcripts, identifying important and/or common themes and compiling an initial list of potential structural and thematic codes. The primary coder [CHA] compiled all potential codes, and the team met multiple times to discuss, review, and revise code definitions, inclusion/exclusion code application criteria, and examples of code applications to representative data. Primary and secondary structural codes were then applied to all transcripts by the secondary coder [CD], maintaining ≥80% ICA with the primary coder. Given the extensive complexities and nuances in the data, the primary coder applied content and thematic codes to all transcripts; the PI [DB], having essential expertise in pediatric oncology, independently labeled all coded text with content and thematic codes on ~25% (n=7) transcripts, maintaining ≥80% ICA with the primary coder via a unitization approach. |
| 27. software: What software, if applicable, was used to manage the data? | NVivo 12 (2018) |
| 28. participant checking: Did participants provide feedback on the findings? | No |
| **REPORTING** | |
| 29. quotations presented: Were participant quotations presented to illustrate the themes / findings? Was each quotation identified? *(e.g., participant number)* | Participant quotations are presented and identified by participant number. |
| 30. data and findings consistent: Was there consistency between the data presented and the findings? | Our manuscript integrates extensive use of direct quotes to provide evidence for each conclusion drawn. |
| 31. clarity of major themes: Were major themes clearly presented in the findings? | Major themes are clearly identified within distinct headings and subheadings. |
| 32. clarity of minor themes: Is there a description of diverse cases or discussion of minor themes? | There is substantial discussion of themes within each subheading, reflecting the diversity of cases. |
